# Immunomodulatory Potential of the Industrialized *Houttuynia cordata* Fermentation Product In Vitro and in Wistar Rats

**DOI:** 10.3390/foods10112582

**Published:** 2021-10-26

**Authors:** Suppawit Utaiwat, Gulsiri Senawong, Kanoknan Khongsukwiwat, Khanutsanan Woranam, Jintana Sattayasai, Thanaset Senawong

**Affiliations:** 1 Department of Biochemistry, Faculty of Science, Khon Kaen University, Khon Kaen 40002, Thailand; suppawitu@gmail.com (S.U.); gulsiri@kku.ac.th (G.S.); yayayha_639@hotmail.com (K.K.); k.woranam@gmail.com (K.W.); 2 Department of Pharmacology, Faculty of Medicine, Khon Kaen University, Khon Kaen 40002, Thailand; sjinta@kku.ac.th; 3 Natural Product Research Unit, Faculty of Science, Khon Kaen University, Khon Kaen 40002, Thailand

**Keywords:** *Houttuynia cordata*, fermentation product, immunomodulation, immunosuppression

## Abstract

*Houttuynia cordata* fermentation products (HCFPs) are produced and widely used as dietary supplements for health and immune support. However, the effect on immune function for these products has not been clearly demonstrated. In this study, soluble fractions of the selected HCFP were used for determination of the immunomodulatory potential, both in vitro and in animal models. Viability and proliferation of rat splenocytes and phagocytic activity of human neutrophils were evaluated. Studies on immunomodulatory effects, including hematological parameters, mitogen-driven lymphocyte proliferation and hemagglutination, were performed in both healthy and immunosuppressed rats. Soluble fraction of the selected HCFP significantly enhanced phagocytic activity of human neutrophils and tended to stimulate splenocyte viability and proliferation. There was no morbidity or mortality for administration of a 14-day regimen of the selected HCFP in both male and female rats. The healthy rats treated with HCFP gained body weight less than the control group, suggesting a reduction in calorie intake. Moreover, low dose of HCFP caused an increased B cell proliferation in ex-vivo, which was related to the increased antibody titer against SRBC in immunosuppressed rats. Our results indicate that the selected HCFP enhances the phagocytic activity of the neutrophils and augments the antibody production in immunosuppressed rats.

## 1. Introduction

The immune system protects our body from environmental invaders and maintains the balance in health. The immunostimulants, including medicines, chemicals, and natural products might improve immune responses through non-specific as well as specific defense mechanisms [1]. The major side effects caused by the frequent use of chemotherapeutic agents for immunomodulation include cytotoxicity to normal cells and immunosuppressive actions [2]. Cyclophosphamide (CTX) is one of the most widely used anticancer agent and a potent immunosuppression drug that can inhibit both humoral and cell mediated immunity [3,4]. CTX is also used to induce immunosuppression in animal models [5,6]. The administration of CTX induces immunosuppression and myelosuppression, which are occasionally life-threatening [7]. Alternatively, there has been a large increase in the number of studies focused on medicinal plants used in various traditional systems [8,9,10]. Herbal medicines and dietary therapies are customarily used as alternative medicine for treatment in conjunction with conventional medicine or after the conventional treatment [9]. Natural products demonstrate an alternative potential to chemotherapies for many illnesses, particularly when the host defense system needs to be activated in immune response [10].

*Houttuynia cordata* Thunb. is a medicinal plant widely distributed in Asia and Southeast Asia. In Thailand, *H. cordata* is mostly found in the Northern and Northeastern regions, and customarily used as a vegetable side dish with local food. *H. cordata* is commonly known as *Plu-khao* or *Khao-tong* in Thailand due to its fishy smell [11]. *H. cordata* has been reported to have several biological activities such as anti-virus [12,13,14,15], anaphylaxis inhibition [16,17], anti-cancer [18,19], anti-allergic [20], and anti-inflammation [21,22,23]. Further, it has also been revealed that *H. cordata* stimulates the immune response. *H. cordata* water extract has been shown to stimulate the proliferation of mouse splenic lymphocytes and T cells in vitro as well as possess anti-SARS activities [24]. *H. cordata* fractions showed valuable therapeutic effects on Th2-mediated (IL-4 and IL-5) or allergic skin disorders [25]. *H. cordata* has a potential role in modulation of innate immune mediators in oral health [26]. Essential oils from *H. cordata* show a potential for growth as well as replacing antibiotics in fish immune responses [27].

Presently, it is believed that fermentation of medicinal plants can promote good health as well as cure diseases [28]. In fact, the fermentation process has been shown to increase flavonoid content [29] as well as the fermented *H. cordata* extract containing identified Bacillus strains from the fermentation process [30]. As mentioned above, fresh *H. cordata* plants have several pharmaceutical activities; however, little is known regarding the pharmaceutical activity of *H. cordata* fermentation products (HCFPs). More specifically, the immunomodulatory activity of the commercial HCFPs available throughout Thailand has not yet been investigated. There are several commercial HCFPs produced by industrialized processes (large-scale productions); however, the product from the Prolac (Thailand) Co., Ltd., Lamphun province, Thailand, was selected for this study based primarily on its availability. Thus, the present study was undertaken to assess in vitro immunomodulatory activity of the selected HCFP through phagocytic activity of human neutrophils and splenocyte viability and to validate its immunomodulatory activity in animal models, both in healthy and cyclophosphamide-induced immunosuppressed rats. The oral toxicity of this product in male and female rats was also established.

## 2. Materials and Methods

### 2.1. Materials

RPMI 1640 medium, fetal bovine serum (FBS), trypsin-EDTA and penicillin/streptomycin from Gibco were purchased from Thermo Fisher Scientific Inc. (Waltham, MA, USA). HiSep™ LSM 1077 was obtained from HiMedia Laboratories Pvt. Ltd. (Mumbai, India). Concanavalin A (Con A), Lipopolysaccharide (LPS) and 3-(4,5-dimethylthiazol-2-yl)-2,5-diphenyltetrazolium bromide (MTT) were purchased from Sigma–Aldrich (St. Louis, MO, USA). Cyclophosphamide monohydrate used as immunosuppressant was purchased from Enzo Life Sciences, Inc. (Farmingdale, NY, USA).

### 2.2. Preparation of HCFP

The *H. cordata* fermentation product (HCFP) was obtained from the Prolac (Thailand) Co., Ltd., Lamphun province, Thailand (batch no. 08042014). The major ingredients of this HCFP are aerial parts of *H. cordata* (993 mg/g) and sugar cane powder (7 mg/g). To remove plant residues in the product, a soluble fraction of HCFP was prepared by centrifugation at 2807× *g* (3500 rpm) for 15 min, 4 °C, then the supernatant was filtered through Whatman No. 4 filter paper (Sigma–Aldrich, St. Louis, USA). The soluble fraction of HCFP was lyophilized by FreeZone Bulk Tray Dryers, Labconco Corporation (Kansas City, MO, USA). After lyophilization, the yields of soluble fraction of HCFP per 1 mL was 13.59 ± 0.71 mg. The soluble fraction of HCFP powder was dissolved to desired concentrations in deionized water for the in vitro study. The soluble fraction of HCFP was also used in the animal study.

### 2.3. In Vitro Phagocytic Activity of Neutrophils

The study using human specimens was reviewed by the Khon Kaen University Ethics Committee for Human Research based on the Declaration of Helsinki and the ICH Good Clinical Practice Guidelines (HE591220). Human peripheral blood was obtained from healthy donors and diluted in Hank’s balanced salt solution (HBSS) buffer without calcium and magnesium ions at 1:1 ratio. Neutrophils were isolated from the blood by HiSep™ LSM 1077, Himedia Laboratories (Mumbai, India) and a density gradient separation technique [31]. Neutrophils was adjusted to 2 × 10^6^ cells/mL in RPMI medium. *Candida albicans* (ATCC 10231) overnight culture was heat killed at 65 °C for 1 h, then harvested by centrifugation, washed three times in PBS. *C. albicans* blastoconidia were resuspended in RPMI medium and diluted to a final concentration of 1 × 10^7^ cells/mL using a hemocytometer. The neutrophils (250 µL, count adjusted to 1 × 10^6^ cells/mL) were incubated with 250 µL of *C. albicans* (count adjusted to 2.5 × 10^6^ cells/mL) in ratio (1:5) and different concentrations of soluble fraction of HCFP (0, 100, 200, 400 and 800 µg/mL) at 37 °C for 30 min in water bath. The system was carefully centrifuged, and the supernatant was discarded. The lower neutrophil layer was resuspended in human serum and smeared on a clean, dry glass slide. The smear was air dried and stained with Giemsa stain. The slides were mounted under a light microscope (BX60, Olympus, Tokyo, Japan) [32]. Two hundred neutrophils per slide were counted. The Candida-engulfed neutrophils were counted as positive cells [33,34]. Phagocytic activity (%) was calculated from the number of positive cells per total 600 neutrophils.

### 2.4. Animals

Male and female Wistar rats, weighing 200–300 g, were purchased from the National Laboratory Animal Center, Mahidol University, Nakhon Pathom, Thailand. Animals were housed under standard environmental conditions of temperature at 23 ± 2 °C under a 12 h-light:12 h-dark cycle and allowed free access to drinking water and standard pelleted diet at Northeast Laboratory Animal Center, Khon Kaen University, Khon Kaen, Thailand. Rats were deprived of food except water 16–18 h prior to the experiments. All experimental protocols in animal experiments were approved by the Animal Ethics Committee of Khon Kaen University (approval ID: AEKKU 17/2557).

### 2.5. In Vitro Splenocyte Viability and Mitogen-Driven Proliferation

#### 2.5.1. Splenocyte Isolation

Splenocytes were isolated from a rat spleen. The healthy 6–7 week old male Wistar rats were sacrificed by CO_2_ inhalation. The rat spleen was aseptically removed and placed into a tube containing PBS with penicillin (100 U/mL) and streptomycin (100 µg/mL). The spleen was pressed using the end of a 3-mL syringe plunger in culture dish containing PBS. The dissociated tissue was gently passed through a cell strainer (70 µm) to produce a suspension of single cells and washed with adding PBS. The cells were collected by centrifugation at 300× *g* for 5 min. The supernatant was carefully removed and discarded without disturbing the pellet. RBC lysis buffer (0.84 % (*w*/*v*) ammonium chloride) was added to cell pellet and re-suspended by shaking the tube. The tubes were incubated in the dark at room temperature for 15 min. After centrifugation, the cell pellet was re-suspended with PBS and collected by centrifugation at 300× *g* for 3 min. After two washes, the cells were finally suspended in complete RPMI-1640 medium. The cells were counted in hemocytometer and the cell viability was determined by trypan blue exclusion method.

#### 2.5.2. MTT Assay

Splenocytes were cultured at 3 × 10^5^ cells/well in 96-wells plates in complete RPMI- 1640 medium in the absence or presence of the mitogens; Concanavalin A (Con A; 5 μg/mL) or Lipopolysaccharide (LPS; 10 μg/mL) [35]. Con A and LPS were added as T-cell and B-cell stimulants, respectively. Different concentrations of soluble fraction of HCFP (0, 100, 200, 400, and 800 µg/mL) were added to each well and the culture plates were incubated at 37 °C in a CO_2_ incubator for 24 and 48 h. After removing old medium, MTT solution (5 mg/mL) and new RPMI medium were added and incubated continuously for 4 h. The formazan was dissolved by adding DMSO (Dimethyl sulfoxide) and incubating for 30 min, the absorbance was read at 550 nm using a microplate reader (EZ Read 2000; Cambridge, UK). The absorbance at 655 nm was used as a reference wavelength. The results were expressed as cell viability (%) and stimulation index (SI) [33,36].

Cell viability (%) = (A_550_-A_655_ of tested sample/A_550_-A_655_ of control) × 100

Stimulation index (SI) = A_550_-A_655_ of tested sample with mitogen/A_550_-A_655_ of control without mitogen

where A is the absorbance.

### 2.6. Dose Selection and Repeated Dose 14-Day Oral Toxicity in Rats

The recommended serving of the commercial HCFP liquid supplement is 5–15 mL twice a day, thus the human equivalent daily dose (HED) is 30 mL (maximum volume) per 60 kg adult. The adult human dose has been converted to animal dose based on body surface area (BSA) normalization method [37] for testing oral toxicity of the selected HCFP in animal model. Male and female Wistar rats were randomly separated to provide 3 rats per group and the HCFP soluble fractions of low and high doses (3.08 and 6.16 mL/kg body weight, respectively) were administered. The rats were continuously administered for 14 consecutive days. After administering doses, animals were observed during the first 30 min, then occasionally during the first 24 h. Body weight was recorded daily. On day 15, animals were humanely killed, and the spleen was immediately removed and weighted. The spleen index was calculated as the spleen weight (mg) per body weight (g).

### 2.7. Immunomodulatory Activity Assays

#### 2.7.1. Antigen Preparation

Sheep red blood cells (SRBC) were used as an antigen to immunize the animals. SRBCs were obtained from fresh sheep blood in Alsever’s solution (1:1) from Salaya Pet Hospital, Nakhon Pathom, Thailand. Sheep blood solution was centrifuged, and the supernatant was removed. SRBC were washed five times with PBS by centrifugation at 450× *g* for 10 min at 4 °C. The washed SRBC were adjusted to a concentration of 10% (*v*/*v*) with normal saline. The animals were immunized by intraperitoneal injection of 0.2 mL of SRBC suspension (10%; *v*/*v*) at day 0 and day 7 of the treatment.

#### 2.7.2. Treatment

Animal experiments were conducted by using both healthy and immunosuppressed rats. The rats were randomly divided into six groups (6 male rats in each group), three groups for healthy rats and three groups for immunosuppressed rats. For immunosuppressed rats, 3 days prior to immunization with antigen, the rats were injected intraperitoneally with CTX (100 mg/kg). On day 0 and day 7, all rats were immunized with 0.2 mL of 10% SRBC. The animals of healthy control and immunosuppressed control groups received drinking water (4 mL/kg); and the treatment groups of low and high doses were treated with 3.08 and 6.16 mL/kg body weight of soluble fraction of HCFP, respectively. All treatments were given orally for 14 days. After 24 h from the last administration, rats were weighed and sacrificed by overdose of Nembutal^®^. The whole blood was collected by cardiac puncture and divided for complete blood counting and hemagglutination assay. The spleen was immediately removed and weighted. The spleen index was calculated as the spleen weight (mg) per body weight (g). The spleen samples were also used for lymphocyte proliferation (ex-vivo).

#### 2.7.3. Complete Blood Count (CBC)

The whole blood was aliquoted into a microcentrifuge tube with EDTA for complete blood count. Total and differential blood cell counts [i.e., white blood cells (WBC), red blood cells (RBC), mean corpuscular hemoglobin (MCH), mean corpuscular hemoglobin concentration (MCHC), hemoglobin (HGB), hematocrit (HCT), mean corpuscular volume (MCV), platelet (PLT), red blood cell distribution width (RDW-SD and RDW-CV), platelet distribution width (PDW), mean platelet volume (MPV), platelet-large cell ratio (P-LCR), plateletcrit (PCT), neutrophils (NEU), lymphocytes (LYM), monocyte (MONO), eosinophil (EO), and basophil (BASO) were assessed by using an automated hematology analyzer (Sysmex Xs-800i), which was serviced by the laboratory of Community Medical Laboratory, Faculty of Associated Medical Sciences, Khon Kaen University.

#### 2.7.4. Determination of Mitogen-Driven Lymphocyte Proliferation (Ex-Vivo)

The spleen samples from each group of treated rats were individually used for lymphocyte proliferation. Splenocytes were cultured at 3 × 10^5^ cells/well in 96-wells plates in complete RPMI 1640 medium in the absence or presence of Con A (5 μg/mL) or LPS (10 μg/mL). The culture plates were incubated at 37 °C in CO_2_ incubator for 48 h. At the end of the incubation period, 10 μL of MTT solution (5 mg/mL) was added to each well and incubation was continued for 4 h. The formazan produced was dissolved by adding 100 μL of DMSO (Dimethyl sulfoxide) to each well. After 30 min incubation, the absorbance was read at 550 nm using a microplate reader (EZ Read 2000; Cambridge, UK). The absorbance at 655 nm was used as a reference wavelength. Responses are expressed as stimulation index (SI): ratio of mean A550–A655 of triplicate cultures with stimulated-mitogen to triplicate cultures without stimulated-mitogen.

#### 2.7.5. Determination of Hemagglutination Assay

Blood samples were collected without anticoagulant from individual animals on day 7 for primary antibody response and on day 14 for secondary antibody response. Serum was separated by centrifugation and antibody titer were determined by the hemagglutination technique. Briefly, the serum was diluted with normal saline by two-fold serial dilution method in a microtitration plate (U bottom). Then, each well was added with 2% SRBC in normal saline and mixed. The plates were then incubated at 37 °C for 1 h and examined for hemagglutination. The reciprocal of the highest dilution of the serum showing 50% agglutination was noted as the antibody titer. The data were presented as the mean ± standard deviation of the mean (SD) of log-2 antibody titer.

### 2.8. Statistical Analysis

Data presented for studies from three independent experiments performed in triplicate were expressed as mean ± standard error of the mean (SEM). Data presented for phagocytic activity and all in vivo studies were expressed as mean ± standard deviation (SD) from three or six rats. The data were compiled and analyzed using GraphPad Prism 6, GraphPad Software (San Diego, CA, USA). One-way ANOVA was employed to assess statistical significance followed by Dunnett’s multiple comparison test.

## 3. Results

### 3.1. Effect of Soluble Fraction (Lyophilized Powder) of HCFP on In Vitro Phagocytic Activity of Human Neutrophils

**Figure 1:**
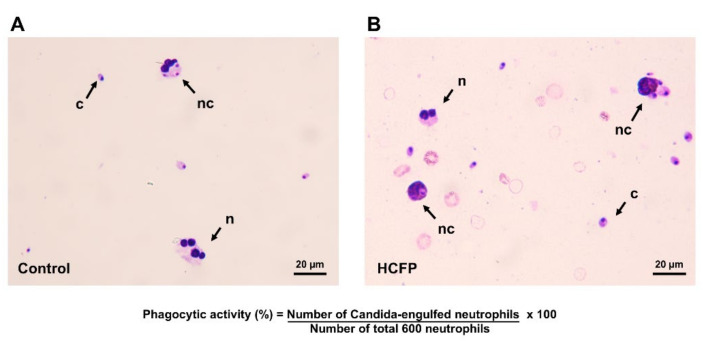
Representative images of phagocytosis of *C. albicans* by human neutrophils. Cells from control—(**A**); deionized water) and HCFP—(**B**); 800 µg/mL) treatments were stained by Giemsa staining and examined under a light microscope. The neutrophils (n) were incubated with *C. albicans* (c) and soluble fraction of HCFP at 37 °C for 30 min. The Candida-engulfed neutrophils (nc) were counted as positive cells. Scale bar = 20 μm.

Phagocytic activity of human neutrophils to engulf *C. albicans* was determined to evaluate the effect of the soluble fraction (lyophilized powder) of HCFP on relative phagocytosis efficiency. The Candida-engulfed neutrophils were observed by using a light microscope (Figure 1). As shown in Table 1, a significant increase in phagocytic activity of neutrophils was observed when incubated with the soluble fraction (lyophilized powder) of HCFP. The phagocytic activity (%) of human neutrophils was increased in the presence of the soluble fraction at concentrations of 200 to 800 µg/mL when compared with a control group (deionized water treated). The result revealed that the soluble fraction of HCFP enhanced phagocytic activity of human neutrophils against *C. albicans*, indicating that HCFP enhanced the innate immune response.

### 3.2. Effect of Soluble Fraction (Lyophilized Powder) of HCFP on In Vitro Splenocyte Viability and Proliferation

**Figure 2:**
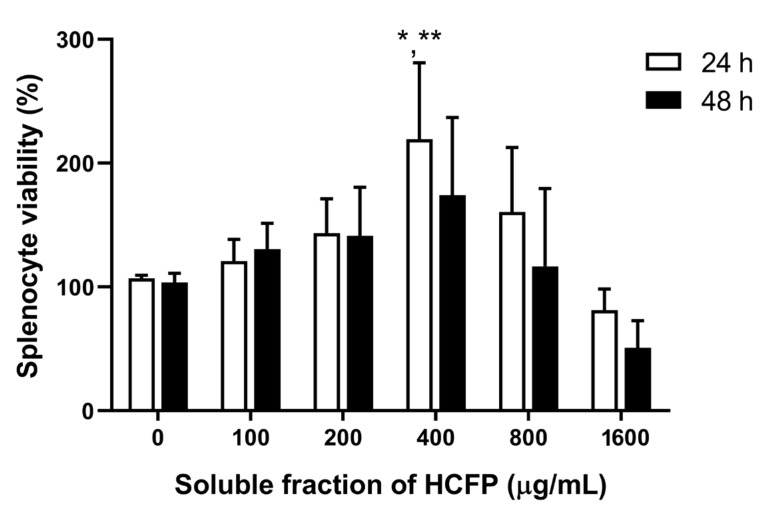
Effect of soluble fraction of HCFP on rat splenocyte proliferation at exposure times of 24 and 48 h. Splenocyte viability (%) was expressed as mean ± SEM from three independent experiments performed in triplicate. * *p* < 0.05 and ** *p* < 0.01 indicate significant differences between different concentrations and control (0 µg/mL). Statistical significance was determined using one-way ANOVA followed by Dunnett’s multiple comparison test.

To determine the effect of the soluble fraction (lyophilized powder) of HCFP on splenocyte viability and proliferation, rat splenocytes were incubated with soluble fraction of HCFP at 0, 100, 200, 400, 800 and 1600 µg/mL. After incubation with soluble fraction of HCFP in the absence of mitogens, proliferation of the rat splenocytes was increased in a dose-dependent manner at concentrations of 100–400 µg/mL (Figure 2). A significant stimulation effect was observed at a concentration of 400 µg/mL for 24 h exposure. The highest concentration of HCFP (1600 µg/mL) caused a decrease in the splenocyte viability at both exposure times of 24 and 48 h, indicating toxicity of high doses of HCFP on rat splenocytes.

**Figure 3:**
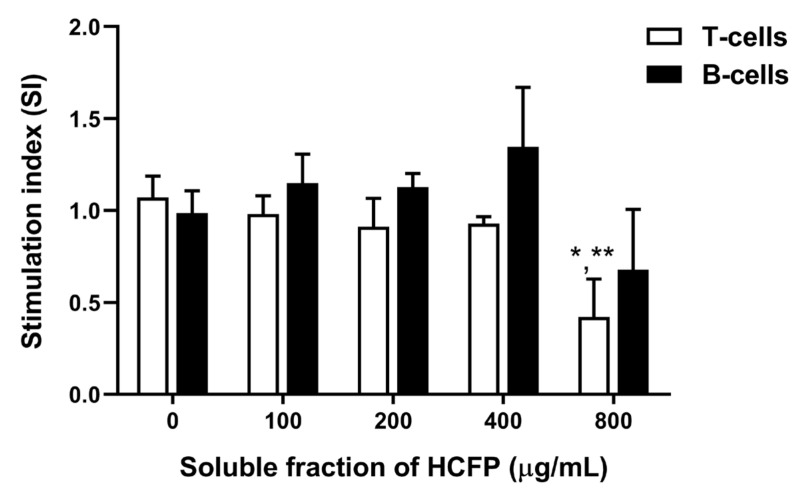
Effect of soluble fraction of HCFP on rat splenocyte mitogen-driven proliferation at 48 h. Stimulation index (SI) was expressed as mean ± SEM from three independent experiments performed in triplicate. * *p* < 0.05 and ** *p* < 0.01 indicate significant differences between different concentrations and control (0 µg/mL). Statistical significance was determined using one-way ANOVA followed by Dunnett’s multiple comparison test.

In the presence of mitogen (Con A or LPS for T-cell or B-cell stimulation, respectively), the effect of HCFP extracts on Con A- and LPS-stimulated splenocyte proliferation was determined. The proliferative responses of splenocytes treated with HCFP were greater than that of the control group in a dose-dependent manner (100–400 µg/mL) for B-cell stimulation with no significant differences (Figure 3). However, HCFP at 800 µg/mL significantly decreased stimulation indexes of splenocytes in T-cell stimulation conditions but not in B-cell stimulation conditions. These results suggest that the soluble fraction of HCFP has the potential to activate B cells involved in antibody production in the humoral immune response.

### 3.3. Oral Toxicity of HCFP in Wistar Rats

The oral toxicity of HCFP soluble fraction was tested in male and female Wistar rats. The tested volume in the rats was calculated from recommended volume of the commercial HCFP consumed per day based on BSA (Body surface area). The body weight and immune organs of the rats were monitored after treatment with different doses of HCFP soluble fraction; the results are shown in Table 2 as the body weight gain (g) and spleen index. The administration of low and high doses (3.08 and 6.16 mL/kg) of HCFP showed no effect on acute oral toxic death in male and female rats. After 14-day oral toxicity treatments, the body weight gains of high dose-treated male rats were lower than that of the control group (*p* < 0.05); however, there was no significant change of the body weight gain in female rats. The spleen index was significantly increased in high dose-treated male rats compared with the control (*p* < 0.05). These results suggested that there were no effects on body weight and immune organs in female rats. However, a high dose of HCFP affected the body weight and immune organ in male rats.

### 3.4. Immunomodulatory Activities of HCFP

#### 3.4.1. Effect of HCFP on Body Weight and Immune Organ of Immunosuppressed Rats

The changes in body weight and immune organs of SRBC-immunized male rats were measured after treatment for 14 days and tabulated (Table 3). The body weights gained in low dose- and high dose-treated healthy rats were significantly decreased when compared with the healthy control group. However, there was no significant change of spleen index in healthy rats treated with HCFP. Treatment with CTX in the immunosuppressed control group showed significant reduction in body weight gain. However, there were no significant changes in body weight gain and spleen index in immunosuppressed rats treated with HCFP.

#### 3.4.2. Effect of HCFP on Hematological Parameters

The hematological changes in both healthy and immunosuppressed rats were determined from the whole blood. The effect of HCFP on hematological parameters in healthy and immunosuppressed rats immunized with SRBC are presented in Table 4. There were no significant changes observed in hematological parameters in healthy rats treated with HCFP at the low and high doses compared with the healthy control group. Comparing between control groups of healthy and immunosuppressed rats, CTX caused decreases in WBC and LYM as well as increases in BASO, RDW-SD, RDW-CV, PDW, MPV, P-LCR, and PCT. Treatment with HCFP produced some changes in the immunosuppressed rats. In the low dose group, reductions in RBC, MONO, HGB, MCHC, PDW, MPV, and P-LCR were observed. In addition, both low and high dose groups showed increases in NEU and EO but decreases in BASO as well as no significant change in LYM (Table 4). These results showed that treatments with HCFP caused no effect on the hematological parameters in healthy rats. However, in immunosuppressed rats, administration of HCFP at high doses significantly changed some hematological values toward the normal levels, indicating the alleviation of immunosuppression.

#### 3.4.3. Effect of HCFP on Mitogen-Driven Lymphocyte Proliferation (Ex-Vivo)

**Figure 4:**
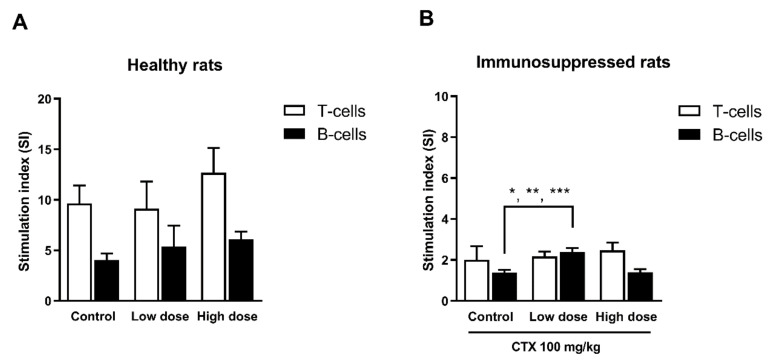
Effect of HCFP on mitogen-driven lymphocyte proliferation (ex-vivo) of T and B cells from healthy rats (**A**) and immunosuppressed rats (**B**) treated for 14 days with different doses of HCFP. Results were expressed in stimulation index (SI) of T and B cells as mean ± SD from six male rats (n = 5, only for high dose group of healthy rats). Control and CTX-control; water administration of 4 mL/kg. Low and high doses; HCFP soluble fraction administration of 3.08 and 6.16 mL/kg, respectively. * *p* < 0.05, ** *p* < 0.01 and *** *p* < 0.001 indicate significant differences between treatments and control group. Statistical significance was determined using one-way ANOVA followed by Dunnett’s multiple comparisons test.

We further investigated the lymphocyte proliferation from the spleen, which is an important step of lymphocyte activation in cell-mediated or humoral immune response. The splenocytes were isolated from treated experimental healthy and immunosuppressed rats. The effect of HCFP on mitogen-driven lymphocyte proliferation of T and B cells is presented in Figure 4. The healthy rats treated with HCFP showed no significant change in both T and B cell proliferations (Figure 4A). However, the immunosuppressed rats treated with the low dose of HCFP showed significantly increased B-cell proliferation (Figure 4B). The enhancing B-cell proliferation activity by HCFP in immunosuppressed rats may involve improvement in the antibody production against SRBC antigen.

#### 3.4.4. Effect of HCFP on Antibody Production of SRBC-Immunized Rats

**Figure 5:**
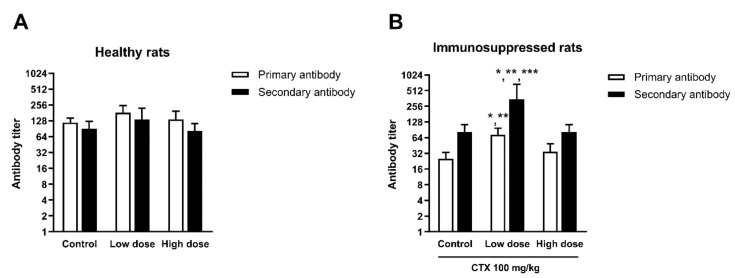
Antibody titers of healthy rats (**A**) and immunosuppressed rats (**B**) treated for 14 days with low and high doses of HCFP. Results of primary and secondary antibodies were expressed in log-2 antibody titers as mean ± SD from six male rats (n = 5, only for High Dose group of healthy rats). Control and CTX-control; water administration of 4 mL/kg. Low and High doses; HCFP soluble fraction administration of 3.08 and 6.16 mL/kg, respectively. * *p* < 0.05, ** *p* < 0.01 and *** *p* < 0.001 indicate significant differences between treatments and control group. Statistical significance was determined using one-way ANOVA followed by Dunnett’s multiple comparisons test.

The effect of HCFP on antibody production against SRBC antigen in healthy and immunosuppressed rats are presented in Figure 5. The results are presented in log-2 antibody titer for both primary and secondary antibodies. The healthy rats treated with HCFP at both low and high doses showed no significant differences in antibody titers when compared with control groups (Figure 5A). However, the immunosuppressed rats treated with the low dose of HCFP showed a significant increase in anti-SRBC antibody titers for both primary and secondary antibodies (Figure 5B). This finding indicates that the soluble fraction of HCFP could enhance the antibody production against SRBC antigen in immunosuppressed rats, indicating a humoral immune response.

## 4. Discussion

In Asian countries, many people believe that fermented plant beverages have the ability to cure most illness [28]. It is anticipated that fermented plant beverages will continue to be popular in the functional food market. The extensive use of industrial HCFPs as a dietary supplement in Thailand without scientific data of their biological properties prompted us to investigate the immunomodulatory potential of the selected commercial HCFP, both in vitro and in animal models. In the in vitro studies, the commercial HCPF was prepared in a soluble fraction. The product has insoluble plant material after the fermentation process. Therefore, the HCFP was filtered to remove the particulates and then used as a soluble fraction. In this study, the yield of soluble fraction per 1 mL of HCFP is a similar quantity to our previous study [38]. A reference chromatographic fingerprint as a typical HPLC chromatogram of the HCFP sample (batch no. 08042014) was obtained and partially identified based on the availability of phenolic acid standards (Supplementary Information Figure S1 and Table S1). The predominant phenolic acid found in the phenolic profile was syringic acid (Table S1), which has been reported to possess potential anti-inflammatory and anti-arthritic effects [39] as well as hepatoprotective effects [40].

In this study, we reported the in vitro immunostimulatory activity of the commercial HCFP for both the innate and adaptive immune responses. Regarding the innate immune response, neutrophils are the first defense white blood cells in innate immunity and our results indicate that the selected HCFP has potential to stimulate the phagocytic activity of neutrophils against foreign antigens. In the adaptive immune response, proliferation of spleen cells is one of the critical steps in the activation of cell-mediated and humoral immune responses [41]. The selected HCFP soluble fraction at concentrations of 100–400 µg/mL showed an ability to stimulate the splenocyte proliferation in a dose dependent manner. However, the selected HCFP at the higher concentrations used (800 and 1600 µg/mL) showed less potent stimulation of splenocyte proliferation than that of the lower concentration used (400 µg/mL). This suggests that the stimulation of splenocyte proliferation may be dependent on specific concentrations. Indeed, exposure to the highest concentration of HCFP (1600 µg/mL) caused a decrease in splenocyte proliferation (Figure 2), indicating that excessive concentrations may inhibit splenocyte proliferation. This may be due to the increased concentration of the toxic ingredient (e.g., aristolochic acid analogues) in high doses of HCFP [42]. Moreover, a significant stimulation effect was observed only for the soluble fraction of HCFP at a concentration of 400 µg/mL for 24 h exposure (Figure 2), suggesting that the stimulation effect may be dependent on exposure times. Splenocytes contain many immune cells, such as T and B lymphocytes, which are involved in lymphocyte activation. To specifically stimulate proliferation of T and B lymphocytes, we exploited the common mitogens [43], concanavalin A (Con A), and lipopolysaccharide (LPS), respectively, for the study. The soluble fraction of HCFP tended to specifically stimulate B lymphocyte proliferation greater than T lymphocyte proliferation (Figure 3). This may be dependent on the type of antigen which is more specific to B lymphocyte.

In the animal study, we firstly tested the oral toxicity of this commercial product in both male and female rats. Low and high doses of the HCFP (3.08 and 6.16 mL/kg) calculated from human administration (30 and 60 mL per 60 kg adult per day, respectively) clearly showed no acute oral toxic death for both male and female rats and caused no effect on body weight gain and spleen index in female rats. However, the high dose of the HCFP caused a decrease in body weight gain in male rats which may be because the HCFP interrupts the food intake or reduces fat accumulation. Regarding this issue, a previous study demonstrated that the ethanolic extract of *H. cordata* noticeably prevented weight gain by reducing fat accumulation and has anti-obesity activity in obese rats without effects on their food intake [44]. Moreover, in the 14-day oral toxicity test, the high dose showed the possibility to improve immunity by increasing the spleen index, which represents an improvement in spleen activity in rats [45]. However, the increase in spleen size observed in this study (Table 2 and Table 3) may also reflect a spleen injury, which would be investigated by histopathological analysis in a future experiment. In SRBC-immunized rats, HCFP also affects rat body weight when compared with the control group. A previous animal study reported that the *H. cordata* water extract was verified as safe with oral administration of 16 g/kg to the laboratory animals [24]. Moreover, the fermented juice of *H. cordata* did not affect the behavioral character, internal organs, body mass, hematological, and biochemical parameters of experimental rats, and the consumption of fermented juice of *H. cordata* was safe to the rodent model system up to the concentration of 9 mL/kg/day [46].

The hematopoietic system is one of the targets for toxicity testing of compounds and plays a key index of physiological and pathological status in human and animal studies [47]. In this study, SRBC was used as a foreign antigen to immunize the rats and the immunomodulatory activity was tested after treatment with HCFP. Animal sensitization to the SRBC antigen primarily develops and spreads to the extravascular space through the lymphatic system and then to the lymph nodes [48]. After treatment with HCFP, there were no different changes in hematological parameters from blood in healthy rats. However, in immunosuppressed rats, treatment with the selected HCFP caused some changes in hematological parameters. CTX causes leucopenia (decrease in WBC, LYM and platelet counts) by alkylation of functional groups in cellular proteins and is also responsible for restraining medulla hematopoietic function [6]. In this study, CTX was used to induce immunosuppression, which caused a decrease in the numbers of WBC and lymphocytes but no significant changes in the platelet count (Table 4). However, CTX caused an increase in BASO, RDW-SD, RDW-CV, PDW, MPV, P-LCR, and PCT. After treatment with HCFP, increasing numbers of neutrophils and eosinophils were observed in immunosuppressed rats. Neutrophils are significant effector cells in the innate immune response [49]. The increased numbers of neutrophils in blood circulation normally occur due to infections and injuries, and the inflammatory reaction also causes an increase in neutrophil levels. Furthermore, the number of eosinophils in blood are shown to increase in specific immune responses, such as allergic diseases [50]. Eosinophils also have a role in sustaining the increased numbers of peripheral B cells, and encouragement of B cell survival, proliferation, and immunoglobulin secretion by a contact-independent mechanism [51].

Proliferation of T and B lymphocytes is a response to specific mitogen stimulation. In this study, the spleens of HCFP-treated healthy and cyclophosphamide-immunosuppressed rats were investigated for the effect of HCFP on lymphocyte proliferation. The HCFP treatment in healthy rats had no significant change for T and B cell proliferations. Interestingly, a low dose of HCFP could stimulate B cell proliferation in cyclophosphamide-immunosuppressed rats (Figure 4B). B cells are one of the crucial components of adaptive immune response. Their differentiation into either specific memory B cells or antibody-secreting plasma cells is a consequence of activation steps that involve the processing and presentation of antigens [52].

Antibody production is a process to specifically protect the body against antigens in a humoral immune response. An antibody acts as the effector of the humoral immune response by binding to the antigens and neutralizing them or eliminating them by cross linking to form clusters, which are digested by phagocytes [53]. Antibody production in response to a specific antigen involves several cellular actions, including antigen processing and presenting, recognition of the presented antigen, and activation and production of cytokines that increase the response of memory B cells. At a first or primary response after exposure to the antigen (SRBC), IgM is initially secreted followed by IgG [48]. After a second exposure to the same antigen, a secondary response is activated and characterized by a rapid and high level of antibody production (nearly all as IgG). In this study, SRBC was used to immunize the rat for production of anti-SRBC antibody, which could be detected by using hemagglutination assay (Figure 5). Primary and secondary responses were significantly raised in HCFP-treated immunosuppressed rats (Figure 5B). According to our findings, the treatment with the selected HCFP improved the hemagglutination antibody titer, suggesting that HCFP may underpin the augmentation of humoral response to SRBC. The increased B cell proliferation and hemagglutination titer observed in this study indicated that HCFP improved and recovered humoral immunity during immunosuppression.

## 5. Conclusions

Our results demonstrated that the commercially produced HCFP exhibited in vitro and in vivo immunostimulatory potentials in both innate and adaptive immune responses. HCFP stimulated phagocytic activity of human neutrophils in vitro and enhanced B lymphocyte proliferation (ex-vivo), which is involved in lymphocyte activation and antibody production in cyclophosphamide-induced immunosuppressed rats. In the animal study, HCFP showed no acute oral toxic death at concentrations tested in both male and female rats. Although HCFP promoted body weight loss in healthy rats, there is no significant change in the immune responses. Moreover, HCFP improved and recovered humoral immunity during immunosuppression. In conclusion, the commercial HCFP may be utilized as a potential immunotherapeutic supplement for enhancing the immune response, especially humoral immunity in immunosuppressed diseases. However, investigations on toxicity due to long term consumption and appropriate doses for safety are still required.

## Figures and Tables

**Table 1:** Effect of soluble fraction (lyophilized powder) of HCFP on phagocytic activity of human neutrophils.

Treatments	Concentrations (µg/mL)	Phagocytic Activity (%)
Control (deionized water)	-	47.74 ± 8.36
Soluble fraction	100	50.47 ± 7.42
	200	68.88 ± 5.36 *
	400	69.22 ± 5.77 *
	800	76.07 ± 6.15 *, **

Phagocytic activity (%) was calculated from the number of Candida-engulfed cells per total 600 neutrophils in each treatment. Phagocytic activity (%) was expressed as mean ± SD from three independent experiments. * *p* < 0.05 and ** *p* < 0.01 indicate significant differences between treatments and control (deionized water). Statistical significance was determined using one-way ANOVA followed by Dunnett’s multiple comparison test.

**Table 2:** Effect of HCFP on body weight and immune organ after 14-repeated day administration in male and female Wistar rats.

Gender	Group	Body Weight Gain (g)	Spleen Index
Male	Control	78.00 ± 17.06	2.54 ± 0.32
	Low dose	101.67 ± 7.10	2.66 ± 0.20
	High dose	32.00 ± 12.73 *	3.37 ± 0.08 *
Female	Control	38.33 ± 5.86	3.38 ± 0.02
	Low dose	26.67 ± 8.51	3.15 ± 0.46
	High dose	27.67 ± 14.01	3.26 ± 0.15

Data were expressed as mean ± SD from three rats for each group of healthy rats. Control; water administration of 4 mL/kg. Low and high doses; HCFP soluble fraction administration of 3.08 and 6.16 mL/kg, respectively. * *p* < 0.05 indicate significant differences between treatments and control group. Statistical significance was determined using one-way ANOVA followed by Dunnett’s multiple comparison test.

**Table 3:** Effect of HCFP on body weight and immune organ after 14-repeated day administration in healthy and immunosuppressed Wistar rats.

	Treatment	Body Weight Gain (g)	Spleen Index
Healthy rats	Control	86.84 ± 5.89	2.51 ± 0.31
	Low dose	68.91 ± 11.43 *	2.46 ± 0.22
	High dose	65.40 ± 13.71 **	2.82 ± 0.42
Immunosuppressed rats	Control	47.71 ± 7.96 ^###^	2.79 ± 0.28
	Low dose	51.39 ± 9.32	2.44 ± 0.35
	High dose	39.46 ± 7.81	2.79 ± 0.21

Data were expressed as mean ± SD from six rats (n = 5 only for High Dose group of healthy rats). Control; water administration of 4 mL/kg. Low and high doses; HCFP soluble fraction administration of 3.08 and 6.16 mL/kg, respectively. * *p* < 0.05 and ** *p* < 0.01 indicate significant differences between treatments and control group. ^###^ *p* < 0.001) indicate significant differences between the control group of healthy rats and control group of immunosuppressed rats. Statistical significance was determined using one-way ANOVA followed by Dunnett’s multiple comparisons test.

**Table 4:** Effect of HCFP on hematological parameters in healthy and cyclophosphamide-induced immunosuppressed rats.

Hematological Parameters	Healthy Rats ^a^			Immunosuppressed Rats ^a^		
	Control	Low Dose	High Dose	Control	Low Dose	High Dose
WBC (10^3^/µL)	5.30 ± 0.73	5.67 ± 0.64	5.53 ± 1.54	3.50 ± 0.66 ^##^	4.21 ± 0.82	4.08 ± 0.86
RBC (10^6^/µL)	8.02 ± 0.64	8.36 ± 0.57	8.07 ± 0.37	7.74 ± 0.32	7.31 ± 0.19 **	7.96 ± 0.13
HGB (g/dL)	14.78 ± 0.98	15.30 ± 1.08	14.96 ± 0.77	14.52 ± 0.56	13.43 ± 0.43 **	14.73 ± 0.33
HCT (%)	45.52 ± 4.06	46.97 ± 4.05	45.66 ± 4.31	44.45 ± 1.68	43.33 ± 1.07	44.43 ± 0.92
MCV (fL)	56.73 ± 1.71	56.15 ± 2.32	56.52 ± 3.14	57.42 ± 1.50	59.27 ± 1.40	55.83 ± 1.09
MCH (pg)	18.45 ± 0.30	18.30 ± 0.30	18.56 ± 0.36	18.73 ± 0.35	18.37 ± 0.23	18.52 ± 0.39
MCHC (g/dL)	32.53 ± 0.97	32.62 ± 0.92	32.86 ± 1.45	32.65 ± 0.33	30.98 ± 0.77 **	33.18 ± 0.29
PLT (10^3^/µL)	754.33 ± 104.40	796.67 ± 217.62	841.80 ± 100.6	845.17 ± 418.56	1061.17 ± 123.6	899.67 ± 281.1
RDW-SD (fL)	27.83 ± 2.08	27.83 ± 1.15	27.68 ± 2.41	34.27 ± 1.68 ^###^	37.68 ± 3.68	32.97 ± 1.52
RDW-CV (%)	15.60 ± 1.60	16.17 ± 1.21	15.70 ± 1.71	19.25 ± 0.69 ^###^	20.15 ± 1.32	19.28 ± 0.84
PDW (fL)	7.28 ± 0.87	7.95 ± 0.71	7.46 ± 0.55	8.60 ± 0.29 ^##^	7.43 ± 0.33 **	8.28 ± 0.41
MPV (fL)	7.43 ± 0.58	7.70 ± 0.39	7.40 ± 0.34	8.22 ± 0.18 ^##^	7.60 ± 0.23 *	8.03 ± 0.022
P-LCR (%)	6.63 ± 3.39	8.25 ± 2.34	6.24 ± 1.74	11.42 ± 1.35^##^	8.38 ± 1.35 **	10.25 ± 1.69
PCT (%)	0.57 ± 0.12	0.61 ± 0.17	0.63 ± 0.09	0.83 ± 0.11 ^#^	0.80 ± 0.08	0.72 ± 0.23
NEU (10^3^/µL)	0.57 ± 0.32	0.74 ± 0.21	0.83 ± 0.39	0.28 ± 0.04	0.55 ± 0.10 *	0.75 ± 0.21 **
LYM (10^3^/µL)	4.40 ± 0.57	4.58 ± 0.48	4.37 ± 1.08	2.65 ± 0.63 ^##^	3.12 ± 0.61	2.94 ± 0.63
MONO (10^3^/µL)	0.13 ± 0.11	0.24 ± 0.09	0.20 ± 0.09	0.09 ± 0.04	0.29 ± 0.07 ***	0.12 ± 0.09
EO (10^3^/µL)	0.12 ± 0.14	0.12 ± 0.04	0.13 ± 0.04	0.09 ± 0.02	0.25 ± 0.12 *	0.23 ± 0.10 *
BASO (10^3^/µL)	0.09 ± 0.22	0.00 ± 0.00	0.00 ± 0.00	0.38 ± 0.12 ^#^	0.00 ± 0.00 **	0.09 ± 0.21 *
NEU (%)	10.43 ± 4.97	13.02 ± 3.13	14.16 ± 4.34	8.33 ± 2.55	13.30 ± 1.91	19.38 ± 4.51 **
LYM (%)	83.28 ± 5.59	80.85 ± 4.02	79.86 ± 4.92	74.83 ± 4.73	74.28 ± 1.74	71.92 ± 4.39
MONO (%)	2.35 ± 1.85	4.10 ± 1.29	3.54 ± 0.96	2.85 ± 1.53	6.73 ± 1.10 ***	2.72 ± 1.68
EO (%)	2.40 ± 3.04	2.03 ± 0.62	2.44 ± 0.27	2.73 ± 0.96	5.68 ± 2.15 *	5.72 ± 2.00 *
BASO (%)	1.53 ± 3.76	0.00 ± 0.00	0.00 ± 0.00	10.97 ± 1.88 ^###^	0.00 ± 0.00 ***	1.77 ± 4.33 **

^a^ Data were expressed as mean ± SD from six male rats (n = 5 only for high dose group of healthy rats). Control; water administration of 4 mL/kg. Low and High doses; HCFP soluble fraction administration of 3.08 and 6.16 mL/kg, respectively. * *p* < 0.05, ** *p* < 0.01 and *** *p* < 0.001 indicate significant differences between treatments and control group. ^#^ *p* < 0.05, ^##^ *p* < 0.01 and ^###^ *p* < 0.001) indicate significant differences between the control group of healthy rats and control group of immunosuppressed rats. Statistical significance was determined using one-way ANOVA followed by Dunnett’s multiple comparisons test.

## Data Availability

The datasets generated and/or analyzed during the study are available from the corresponding author upon reasonable request.

## Supplementary Materials

The following are available online at https://www.mdpi.com/article/10.3390/foods10112582/s1, Figure S1: HPLC chromatograms of phenolic acid standards (A) and HCFP soluble fraction (B), Table S1: Phenolic acid compositions of the soluble fraction of HCFP in µg/g.
