# Supplementary material for: Immunomodulatory Potential of the Industrialized Houttuynia cordata Fermentation Product In Vitro and in Wistar Rats
Source: Foods. 2021 Oct 26;10(11):2582. doi: 10.3390/foods10112582 (PMC8619134; doi:10.3390/foods10112582)
Supplement: Supplementary file 1 [file foods-10-02582-s001.zip › foods-1422198-supplementary.pdf]

Supplementary

Immunomodulatory Potential of the Industrialized *Houttuynia cordata* Fermentation Product In Vitro and in Wistar Rats

Suppawit Utaiwat [1], Gulsiri Senawong [1], Kanoknan Khongsukwiwat [1], Khanutsanan Woranam [1], Jintana Sattayasai [2] and Thanaset Senawong [1,3,*]

[1] Department of Biochemistry, Faculty of Science, Khon Kaen University, Khon Kaen 40002, Thailand; suppawitu@gmail.com (S.U.), gulsiri@kku.ac.th (G.S.), yayayha_639@hotmail.com (K.K.), k.woranam@gmail.com (K.W.)
[2] Department of Pharmacology, Faculty of Medicine, Khon Kaen University, Khon Kaen 40002, Thailand; sjinta@kku.ac.th
[3] Natural Product Research Unit, Faculty of Science, Khon Kaen University, Khon Kaen 40002, Thailand
* Correspondence: sthanaset@kku.ac.th

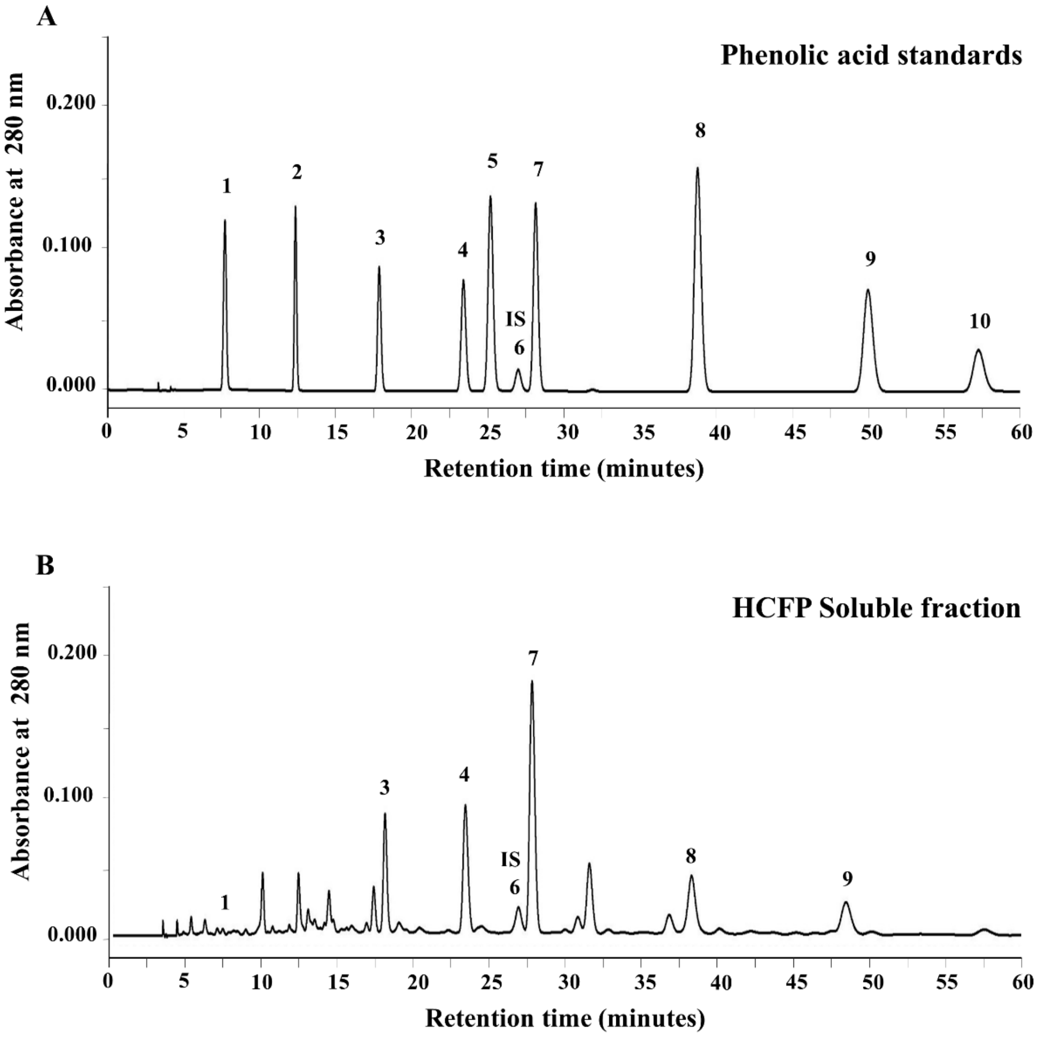

Figure S1. HPLC chromatograms of phenolic acid standards (**A**) and HCFP soluble fraction (**B**), where 1= gallic acid, 2 = protocatechuic acid, 3 = *p*-hydroxybenzoic acid, 4 = vanillic acid, 5 = caffeic acid, 6 = *m*-hydroxybenzaldehyde, 7 = syringic

acid, 8 = *p*-coumaric acid, 9 = ferulic acid and 10 = sinapinic acid. The *m*-hydroxybenzaldehyde was used as an internal standard (IS).

Table S1. Phenolic acid compositions of the soluble fraction of HCFP in μg/g.

Phenolic acids	Soluble fraction of HCFP (μg/g)
Gallic acid	14.36 ± 4.96
p-Hydroxybenzoic acid	64.30 ± 5.68
Vanillic acid	79.97 ± 11.30
Syringic acid	88.23 ± 12.83
p-Coumaric acid	20.53 ± 0.94
Ferulic acid	32.22 ± 4.94
